# Successful management of left ventricular high lateral wall rupture during bentall procedure: a case report

**DOI:** 10.3389/fcvm.2026.1759056

**Published:** 2026-02-09

**Authors:** Ke Xu, Lailong Sun, Yi Li, Songlin Zhang

**Affiliations:** Department of Thoracic and Cardiovascular Surgery, First Clinical Medical College of China Three Gorges University & Yichang Central People’s Hospital, Yichang, Hubei, China

**Keywords:** aortic valve insufficiency, arrhythmia, bentall procedure, combined intracardiac and extracardiac repair, ventricular rupture

## Abstract

Left ventricular rupture is a highly fatal complication, with reported in-hospital mortality rates as high as 80%, largely due to the challenge of achieving timely surgical intervention. We present the case of a 59-year-old female who underwent a Bentall procedure for moderate-to-severe aortic regurgitation with concomitant aortic sinus and ascending aortic dilation. Shortly after weaning from cardiopulmonary bypass, a sudden rupture of the high lateral left ventricular wall occurred. Successful management involved a combined intracardiac and extracardiac repair: the external tear was first exposed, followed by exploration to identify and suture the corresponding internal defect. Over a 4-year postoperative follow-up, the patient maintained good cardiac function with no cardiac-related mortality. This case highlights the critical therapeutic significance of combined intracardiac and extracardiac repair for left ventricular rupture and underscores key technical lessons for managing this catastrophic event.

## Introduction

The Bentall procedure is the established gold standard for surgical management of aortic root pathologies, including aortic sinus dilation, aneurysm, and type A aortic dissection (1). Despite its life- saving role, the complexity of the procedure entails a risk of rare but severe complications. Among these, intraoperative left ventricular rupture—especially when it involves the deep, poorly accessible upper lateral wall- represents a critical challenge even for experienced surgeons. Such rupture typically occurs abruptly, leading to torrential hemorrhage and refractory cardiogenic shock, with extremely low rescue success rates and scant documentation in the literature. Reported data indicate that left ventricular free wall rupture (LVFWR), one of the most fatal cardiac events, carries an intraoperative mortality of 40% and an in- hospital mortality as high as 80% (2), most commonly occurring in hypertensive patients older than 55 years (3). To date, no comprehensive literature has systematically addressed LVFWR occurring specifically during aortic root surgery. While surgical repair under cardiopulmonary bypass remains the mainstay of treatment (4), specific strategies for managing rupture in this context are not well defined.

Here, we report a case of sudden high lateral wall rupture of the left ventricle immediately after weaning from cardiopulmonary bypass during a Bentall procedure. We describe the potential precipitating factors, the intraoperative decision-making process, and the technical details of a combined cardiothoracic and intracardiac repair. This report aims to share a successful management experience, summarize key lessons, underscore the therapeutic importance of a intracardiac and extracardiac repair for left ventricular rupture, and propose a structured approach for handling this devastating complication.

## Case presentation

**Figure 1:**
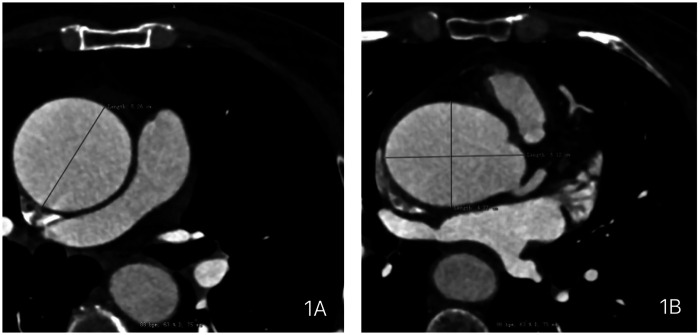
Preoperative chest computed tomography (CT) of the patient demonstrating: **(A)** dilated ascending aorta with a maximum diameter of 5.26 cm. **(B)** Dilated aortic sinus with dimensions of 6.12 × 4.77 cm.

**Figure 2:**
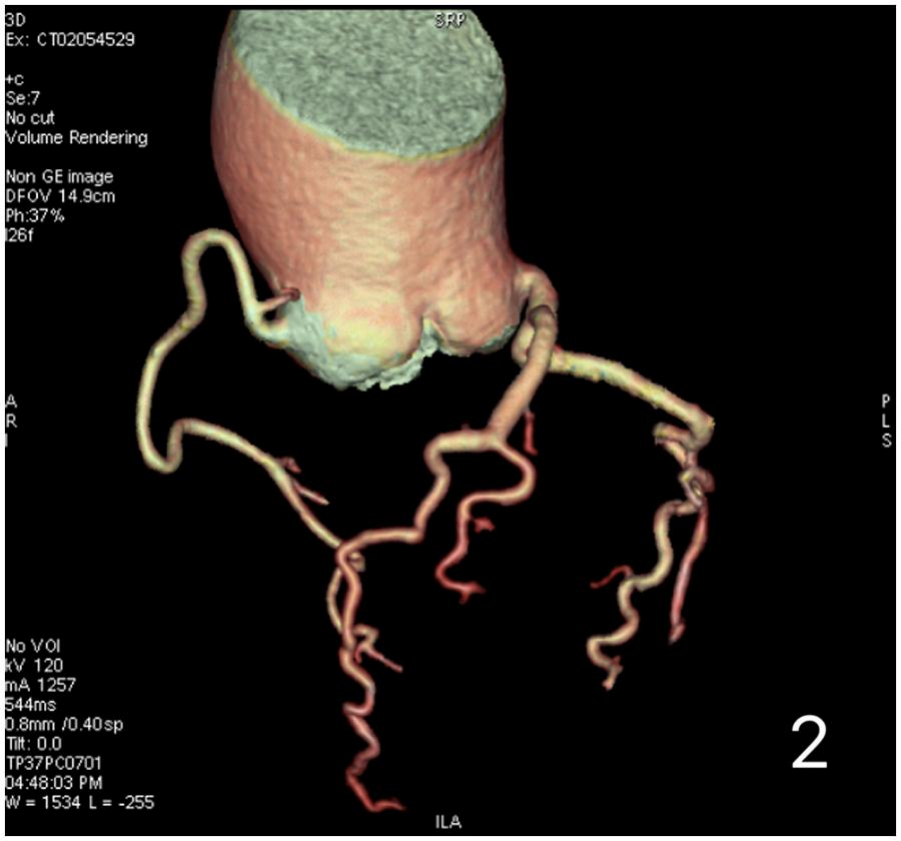
Preoperative coronary computed tomography angiography (CTA) of the patient demonstrates no significant coronary artery stenosis.

A 59-year-old female presented with a 3-year history of chest tightness and palpitations following exertion, which resolved with rest. Over the past six months, her symptoms had progressed in severity, accompanied by shortness of breath, fatigue, and dizziness. Transthoracic echocardiography (TTE) at our institution revealed: Valvular heart disease: Moderate-to-severe aortic regurgitation.Aortic pathology: Annulo-aortic ectasia. Chamber abnormality: Left ventricular enlargement. Ventricular function: Normal left ventricular systolic function with impaired diastolic function (Left ventricular end-diastolic diameter: 55 mm, Left ventricular ejection fraction: 63%, Aortic diameter: 51 mm, Aortic annulus diameter: approximately 30 mm, Aortic sinus diameter: approximately 43 mm). Computed Tomography (CT) examinations showed: Non-contrast Chest CT: widened aorta (Figures 1A,B). Coronary Computed Tomography Angiography (CTA): Right-dominant coronary circulation. A myocardial bridge in the mid-segment of the left anterior descending (LAD) artery. Aortic dilatation (Figure 2).

The patient was diagnosed with: (1) Moderate-to-severe Aortic Regurgitation; (2) Aortic sinus and ascending aorta dilatation; (3) Occasional Atrioventricular Premature Contractions.

Following standard preoperative evaluation and exclusion of contraindications, the patient underwent a Bentall procedure. A 25-mm valved conduit was employed, involving resection of the native aortic valve, implantation of the composite graft, and reimplantation of the left and right coronary arteries as buttons. The distal end of the graft was anastomosed to the transected ascending aorta.

After de-airing and releasing the aortic cross-clamp, systemic rewarming was initiated and cardiopulmonary bypass was weaned. Protamine sulfate was administered for heparin reversal. Upon termination of bypass, however, massive arterial bleeding was noted within the pericardial well. Exploration identified a rupture in the high lateral wall of the left ventricle.

Cardiopulmonary bypass was urgently re-established with systemic heparinization. The aorta was re-clamped, and cardioplegia was administered for myocardial protection. The left ventricular rupture was fully exposed by dissecting from the external tear toward the endocardial orifice. The defect was repaired using a combined approach: both the internal and external orifices were closed with a continuous 4-0 polypropylene suture, buttressed with a polyester patch and an autologous pericardial patch, respectively. The repair was further reinforced with biological glue.

The patient was rewarmed, the aortic cross-clamp was removed, and the heart resumed spontaneous sinus rhythm. Inspection confirmed hemostasis at the repair site. Cardiopulmonary bypass was successfully discontinued, and the cannulas were removed. After ensuring meticulous hemostasis, mediastinal and pericardial drains were placed, and the chest was closed in a standard fashion.

**Figure 3:**
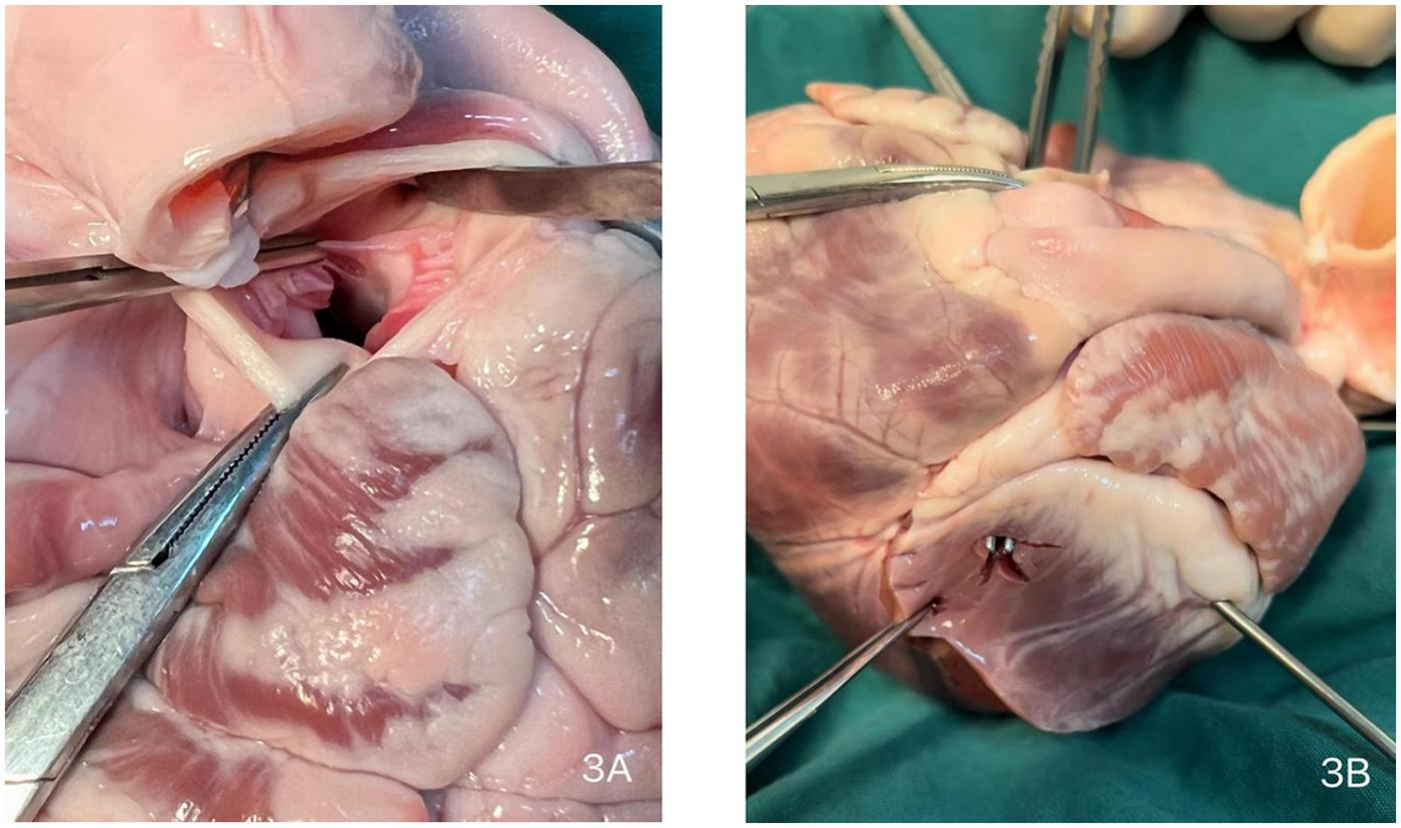
Intraoperative schematic diagram. **(A)** Site of aortic valve replacement. **(B)** Location of the left ventricular high lateral wall rupture.

**Figure 4:**
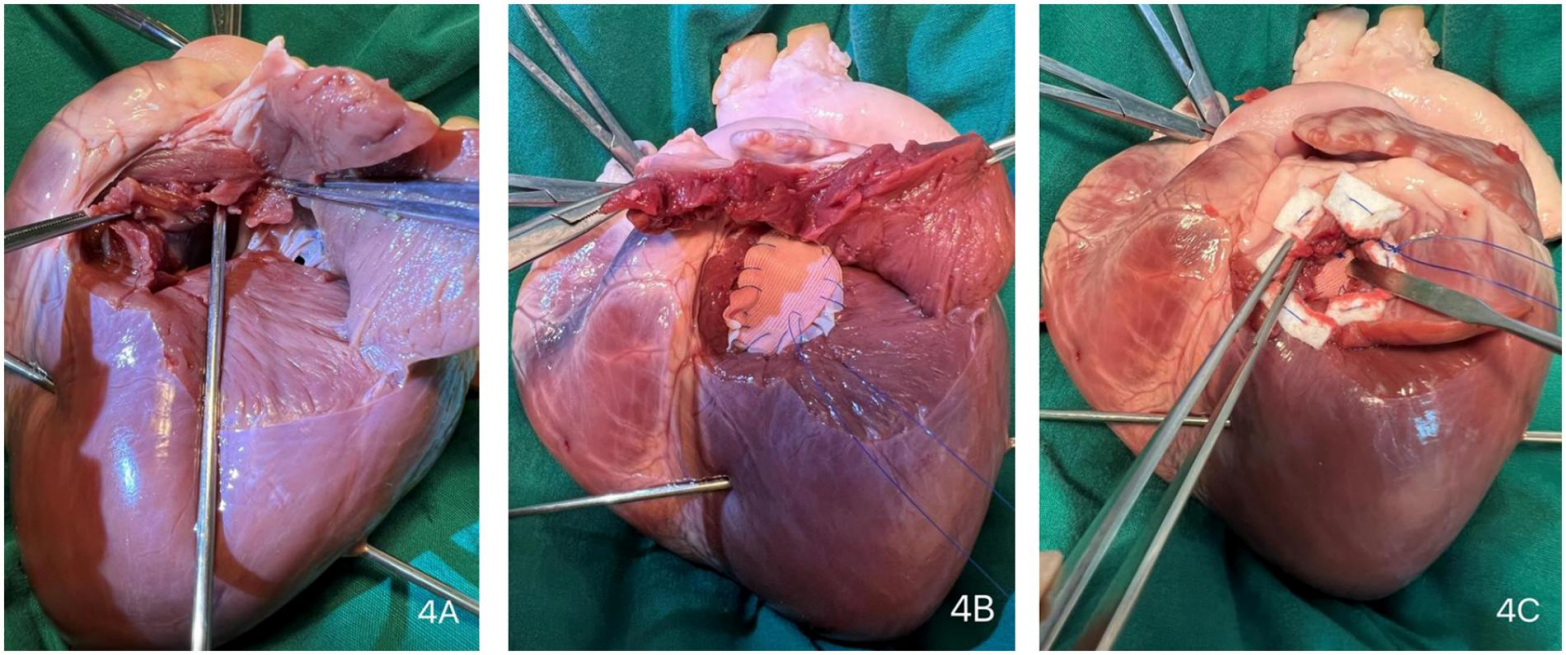
Surgical repair procedure. (Extensive dissection ensures thorough exposure of the surgical site, obviating the need for large-scale anatomical resection during the procedure) **(A)** Parallel to the left anterior descending (LAD) coronary artery, the external orifice was enlarged to explore the internal orifire. **(B)** The internal orifice was closed using a continuous suture with a polytetrafluoroethylene (PTFE) felt strip lined with an autologous pericardial patch. **(C)** An external PTFE felt patch was applied to secure the internal patch firmly against the endocardium.

Due to the critical nature of the intraoperative event, no photographic documentation was obtained during the actual emergency repair. The surgical scenario was subsequently simulated and demonstrated using a porcine heart model (Figures 3, 4, and Supplementary Video S1).

The patient was transferred to the intensive care unit (ICU) postoperatively with an endotracheal tube in place. Extubation was successfully performed on postoperative day (POD) 1. She was transferred from the ICU to the general ward on POD 9 and was subsequently discharged home in a stable condition on POD 19.The patient has been followed up for 4 years and remains in good clinical condition. The most recent follow-up evaluations revealed the following:Electrocardiogram: Showed normal sinus rhythm.Transthoracic Echocardiogram:Status post mechanical aortic valve replacement: The prosthetic valve was functioning normally. Segmental wall motion abnormality. Reduced left ventricular systolic function(Left ventricular end-diastolic diameter: 53 mm, Left ventricular ejection fraction: 48%, Aortic diameter: 27 mm).

## Discussion

This report describes a rare case of left ventricular high lateral wall rupture occurring during a Bentall procedure. Cardiac rupture is not a typical complication of this surgery, and the location of the rupture in this case holds significant clinical implications. The standard Bentall procedure primarily focuses on the aortic root and left ventricular outflow tract (5, 6). The high lateral wall of the left ventricle, however, is anatomically remote from this operative field. Supplied by the left circumflex coronary artery and adjacent to the mitral apparatus, this area is not typically involved in direct surgical manipulation. Consequently, the mechanism of rupture in this location is more likely related to indirect factors—such as ventricular distension due to inadequate myocardial protection, pre-existing local myocardial pathology (e.g., old infarction), or rare coronary injury—rather than direct surgical trauma. Given the patient's normal preoperative coronary CTA, absence of regional wall motion abnormalities, and no electrocardiographic evidence of myocardial infarction, an ischemic etiology appears unlikely in this case. Consequently, inadvertent intraoperative injury to the left ventricular wall remains a plausible consideration that cannot be excluded.

Reported cases of cardiac rupture in Bentall procedures are often technically related and localized to the proximal (ventricular) anastomotic site of the composite graft. These include post-operative pseudoaneurysm formation (7), anastomotic dehiscence (8), traumatic rupture (9), and posterior left ventricular outflow tract rupture (10). Proposed mechanisms include: (1) Deep suturing: posterior sutures penetrating too deeply during the annular anastomosis, injuring the underlying left ventricular myocardium; (2) Tissue fragility: inherent weakness of the myocardial and aortic wall in patients with connective tissue disorders like Marfan syndrome or degenerative changes (11); (3) Over-aggressive decalcification: excessive debridement of a severely calcified aortic valve, potentially undermining the subannular myocardial support; and (4) Issues related to coronary reimplantation: although not a direct cause, malalignment or excessive tension on the coronary “buttons” may induce myocardial ischemia, indirectly elevating the risk of rupture.

In contrast,high lateral wall rupture is more suggestive of indirect or non-technical etiologies. These primarily encompass: (1) Ventricular chamber over-distension: Acute dilation of the left ventricle upon weaning from cardiopulmonary bypass, potentially due to increased afterload or myocardial dysfunction, can lead to a critical rise in wall stress and rupture at its weakest point, such as the high lateral wall; (2) Old myocardial infarction: Scar tissue from a prior infarction in the lateral wall significantly reduces myocardial tensile strength, predisposing it to spontaneous rupture even under normal surgical conditions (12); (3) Cardiac adhesions: In re-operative surgery, mechanical traction on the lateral wall during adhesiolysis can cause injury; and (4) Coronary artery injury: Accidental compromise or distortion of the left circumflex artery during coronary button reimplantation can cause acute lateral wall ischemia and subsequent rupture.

The present case involved a patient undergoing first-time cardiac surgery. Preoperative echocardiography and coronary angiography did not indicate left ventricular hypertrophy, myocardial fibrosis, or pre-existing myocardial weakness secondary to chronic hypertension. Based on intraoperative findings and anatomical analysis, we suspect the rupture may have resulted from excessive ventricular dilatation combined with deep suture placement during the aortic annular anastomosis, where posterior sutures could have penetrated too deeply and injured the underlying left ventricular myocardium.

Upon encountering sudden torrential hemorrhage during the procedure, we systematically traced the extracardiac rupture to localize and expose the corresponding intracardiac defect, which was confirmed to be situated on the high lateral wall of the left ventricle. In evaluating repair options, we recognized that while a patch-based sutureless approach is effective for controlling hemorrhage in apical ruptures, it carries a documented risk of postoperative pseudoaneurysm formation (13). Conversely, simple external suturing was deemed likely to propagate the tear (14). Given that patch repair provides superior tension distribution and is particularly suited to complex myocardial disruptions (15), we integrated these principles by employing a simultaneous intracardiac and extracardiac dual-plane repair strategy, which was successfully executed to achieve definitive hemostasis and anatomical closure.

This case underscores the critical importance of “precise suturing” as an unwavering principle in the Bentall procedure (16). Specifically, during the posterior annular anastomosis, the surgeon must have a precise understanding of the patient's individual anatomy from preoperative imaging and must remain vigilant regarding suture depth, avoiding full-thickness bites that could injure the underlying ventricle. Furthermore, intraoperative transesophageal echocardiography should be utilized as a vital tool for real-time assessment of cardiac structure and function, emphasizing a team-based approach to ensure procedural safety.

## Conclusion

In summary, rupture of the high lateral wall of the left ventricle during Bentall surgery, though exceedingly rare, represents a possible and serious complication. Unexplained intra- or postoperative hemorrhage should prompt immediate surgical exploration and timely hemostatic intervention. Given variations in patient baseline status and corresponding surgical approaches, conventional direct suturing may prove ineffective and risk extending the tear, as observed in this case. A systematic approach to assessing the rupture site, combined with a coordinated intracardiac and extracardiac repair strategy, provides valuable technical guidance and supports clinical decision-making in the management of such complex scenarios.

## Data Availability

The original contributions presented in the study are included in the article/Supplementary Material, further inquiries can be directed to the corresponding author.
