# Supplementary figures and images for: Successful management of left ventricular high lateral wall rupture during bentall procedure: a case report
Source: Front Cardiovasc Med. 2026 Feb 9;13:1759056. doi: 10.3389/fcvm.2026.1759056 (PMC12926444; doi:10.3389/fcvm.2026.1759056)

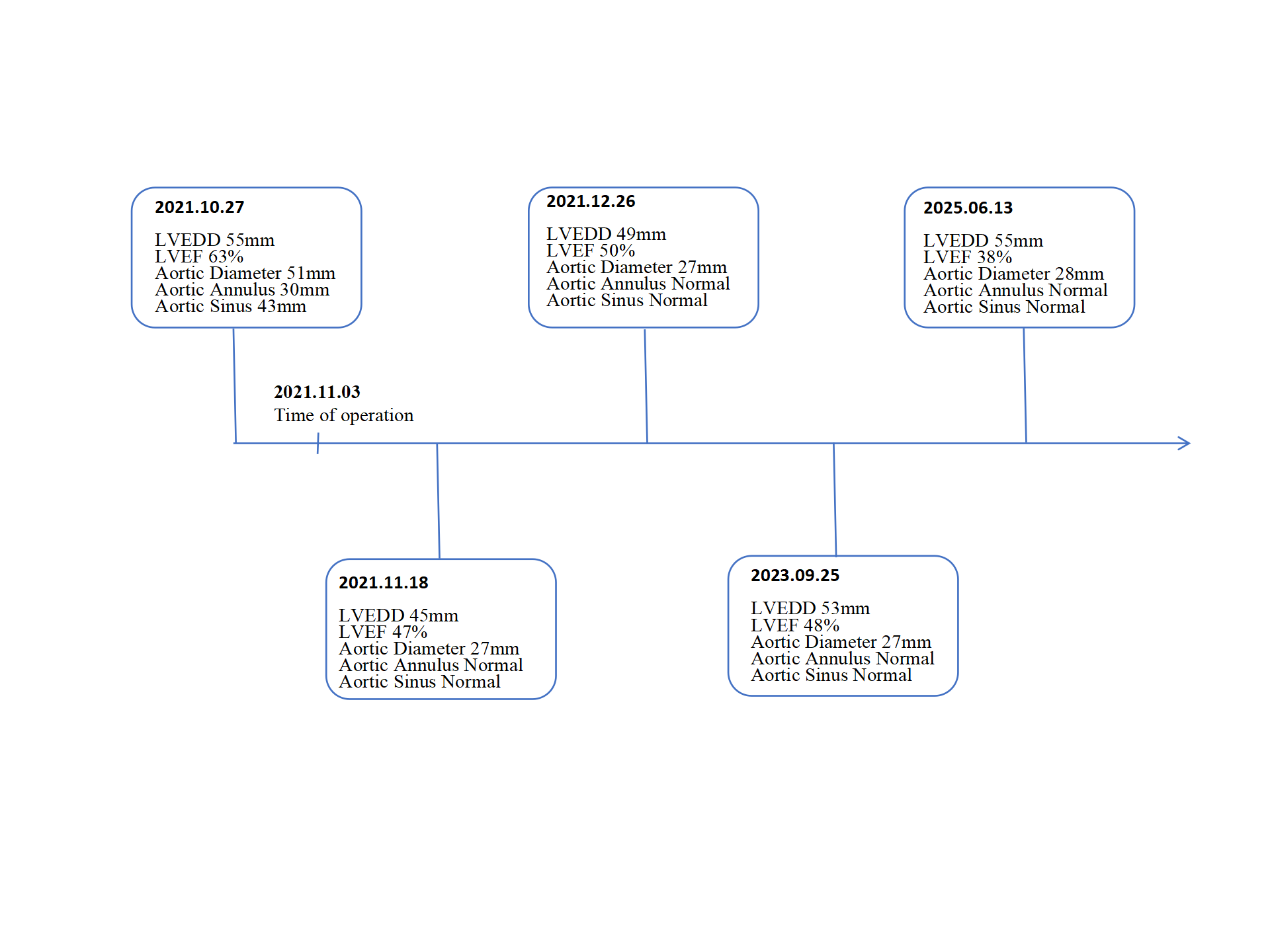

Supplement: Supplementary file 2 [file Image1.tif]
